# The association between liver enzymes and risk of type 2 diabetes: the Namwon study

**DOI:** 10.1186/1758-5996-6-14

**Published:** 2014-02-06

**Authors:** Hye-Ran Ahn, Min-Ho Shin, Hae-Sung Nam, Kyeong-Soo Park, Young-Hoon Lee, Seul-Ki Jeong, Jin-Su Choi, Sun-Seog Kweon

**Affiliations:** 1 Department of Preventive Medicine, Chonnam National University Medical School, 5, Hak-dong, Dong-gu, Gwangju 501-746, Korea; 2 Department of Preventive Medicine and Public Health, School of Medicine, Chungnam National University, Daejun, Korea; 3 Department of Preventive Medicine, College of Medicine, Seonam University, Namwon, Korea; 4 Department of Preventive Medicine & Institute of Wonkwang Medical Science, Wonkwang University school of Medicine, Iksan, Korea; 5 Department of Neurology & Research Institute of Clinical Medicine, Chonbuk National University Medical School & Chonbuk National University Hospital, Jeonju, Korea

**Keywords:** Chronic disease, Diabetes, Epidemiology

## Abstract

**Background:**

We examined the association between liver enzymes and development of type 2 diabetes in a general Korean population.

**Methods:**

A total of 10,667 subjects (4,201 males and 6,466 females) aged 45 to 74 years participated in a baseline examination between 2004 and 2007. Among the subjects, 8,157 (3,231 males and 4,926 females) underwent follow-up examination from 2007 to 2011, for a median follow-up period of 4.2 years. Type 2 diabetes was defined as intake of anti-diabetic agents, insulin treatment, fasting glucose concentration of more than 126 mg/dl, or hemoglobin A1c of more than 6.5% at re-examination. Associations of liver enzymes with incidence of type 2 diabetes were analyzed using logistic regression models.

**Results:**

During the follow-up period, 548 subjects (235 males, 313 females) developed type 2 diabetes. After adjusting for comprehensive diabetes risk factor, the risk of type 2 diabetes was significantly higher in the highest alanine aminotransferase (ALT) quartile than in the lowest quartile (odds ratio (OR): 1.95, 95% confidence interval (CI): 1.18-3.21 in males; OR: 1.49, 95% CI: 1.03-2.16 in females). Similar results were observed for gamma-glutamyltransferase (GGT) quartiles, but in the fully adjusted analysis, the OR for the highest versus lowest quartiles was significant only for females (OR: 1.58, 95% CI: 0.95-2.63 in males; OR: 1.85, 95% CI: 1.23-2.79 in females).

**Conclusions:**

Our results suggest that serum ALT concentrations were independently associated with type 2 diabetes in both sexes, and that GGT was also independently associated but only in females.

## Background

The liver plays an important role in maintenance of normal glucose levels during fasting as well as in the postprandial period [1], and its role in the pathogenesis of type 2 diabetes has attracted much interest. Indeed, hepatic dysfunction resulting from insulin-resistance syndrome may lead to development of type 2 diabetes [2].

The liver enzymes, aspartate aminotransferase (AST), alanine aminotransferase (ALT), and gamma-glutamyltransferase (GGT), are routinely used in evaluation of liver function. Aspartate aminotransferase (AST) and ALT are considered markers of hepatocellular health, whereas GGT also indicates biliary tract function. Alanine aminotransferase (ALT) is the most specific marker of liver pathology and is found primarily in this organ [3]. AST and GGT are also found in other tissues and are therefore less specific markers of liver function [4]. Although GGT is a less specific marker of liver function, higher GGT levels have been found to be an independent predictor of the incidence of type 2 diabetes.

A number of prospective studies have examined the associations between concentrations of AST [5-12], ALT [5-11,13,14], or GGT [3,4,6-11,14-21] and the incidence of type 2 diabetes. A few studies have examined the association of serum AST with risk for type 2 diabetes; two of these reported a significant association between serum AST and diabetes after adjustment for potential confounders [5,9]. Most previous studies examining the association between liver enzymes and incidence of type 2 diabetes have included only ALT or GGT. A limited number of prospective studies have examined the associations of AST, ALT, and GGT with risk of type 2 diabetes [6-14]; however, the results were inconsistent.

The purpose of this study was to examine the associations of serum liver enzyme (AST, ALT, and GGT) levels on the incidence of type 2 diabetes in a community-based prospective cohort study of Korean individuals aged 45 to 74 years.

## Methods

### Study population

Data were derived from the Namwon Study [22]. The Namwon Study is a prospective, population-based cohort study conducted in the city of Namwon, Jeollabukdo, Korea. The study targets cardiovascular disease, osteoporosis, and dementia in elderly Koreans. The 2005 population census performed by the Korean National Statistics Office reported 33,068 residents (14,960 males and 18,108 females) aged 45–74 in Namwon city. From 2004 to 2007, all eligible residents aged 45–74 were invited to participate by mail and telephone, based on the list of officially registered residents. A total of 10,667 subjects (4,201 males and 6,466 females; response rate 32.3%) underwent clinical examinations following questionnaire interviews.

Of the 10,667 subjects, 8,157 (3,231 males and 4,926 females; response rate 76.6%) participated in follow-up examinations between 2007 and 2011. Follow-up examinations were conducted using the same protocol as the baseline study. Of these 8,157 subjects, 1,231 (15.1%) were excluded: 935 (11.5%) subjects with type 2 diabetes at baseline examination; 146 (1.8%) subjects who were diagnosed by physicians with chronic hepatitis B or C, liver cirrhosis, or liver cancer; 89 (1.1%) subjects with incomplete data on any of the covariables; and 61 (0.7%) subjects without a blood sample at baseline or follow-up examination. Finally, the prospective analysis included 6,926 subjects (2,603 males and 4,323 females) aged 45–74 years at baseline examination. Incidence of type 2 diabetes was identified as intake of anti-diabetic agents, receiving insulin treatment, fasting glucose levels ≥126 mg/dl, or hemoglobin A1c levels ≥6.5% at follow-up examination.

### Baseline assessment

All subjects underwent a clinical examination and answered a questionnaire, which included questions on lifestyle, medical history (including diabetes, hypertension, and other chronic diseases), and medical treatment with insulin and oral anti-diabetic agents.

Subjects were classified into three groups according to their alcohol intake status: nondrinkers, ex-drinkers, and current drinkers. Subjects were characterized on the basis of smoking status as never smokers, ex-smokers, and current smokers. Physical activity was classified into two categories according to whether subjects reported engaging in any physical activity, such as jogging, bicycling, swimming, or climbing.

Trained medical personnel performed anthropometric measurements. Body mass index (BMI), calculated as weight divided by the squared height in meters, was used as an index of relative weight. Waist circumference (WC) was measured to the nearest 0.1 cm using a tapeline. Blood samples were obtained from an antecubital vein in the morning after a 12-h overnight fast. Serum was separated on site within 30 min and was stored at − 70°C until analysis. AST, ALT, GGT, total cholesterol (TC), high-density lipoprotein cholesterol (HDL-C), and triglycerides (TG) levels were measured by an automatic analyzer (HITACHI-7600, Hitachi, Japan). The HbA1c levels were analyzed by high-performance liquid chromatography (HPLC) using the VARIANT II system (Bio-Rad, Hercules, CA, USA). Serum C-reactive protein (CRP) was measured by latex-enhanced nephelometry by high-sensitivity assays on the Behring Nephelometer II analyzer (Dade-Behring Diagnostics, Marburg, Germany). The index of insulin resistance was calculated on the basis of fasting values of plasma glucose and insulin, according to the homeostasis model assessment (HOMA) method [23], as insulin resistance (%) = [insulin (units/ml) × fasting glucose (mmol/l)]/22.5. All subjects had provided written informed consent for participation in the study. The Chonnam National University Hospital Institutional Review Board approved this study.

### Statistical analyses

The period of time before the follow-up examination was calculated as the interval between the baseline examination and re-examination. Baseline data are presented as means ± standard deviation (SD) or percentages for categorical variables according to diabetes status at follow-up. Baseline differences in general and biochemical variables were compared using the *t*-test (Mann–Whitney U test for AST, ALT, GGT, TG and CRP) or *chi*-square test for categorical variables. Associations of liver enzymes with the incidence of type 2 diabetes were analyzed using logistic regression models. Analyses were performed separately for males and females. The levels of liver enzymes were divided into four groups, using sex-specific 25th, 50th, and 75th percentiles as cut-points: for AST, 21, 25, and 31 U/L in males and 19, 22, and 26 U/L in females; for ALT, 16, 21, and 30 U/L in males and 14, 17, and 22 U/L in females; for GGT, 20, 31, and 55 U/L in males and 13, 16, and 22 U/L in females. Four models were constructed for each liver enzyme: the first model included age; the second model included all previous factors plus BMI, WC, TC, HDL-C, log TG, alcohol intake status, smoking status, physical activity, and follow-up period; the third model included all previous factors plus log CRP; and the fourth model included all previous factors plus fasting glucose and the HOMA value. Because the distributions of AST, ALT, GGT, TG and CRP were skewed, we log-transformed these values prior to inclusion in a logistic regression model. Interactions of ALT and GGT with BMI, WC, and alcohol intake status were estimated using a logistic regression model. Linear trends were evaluated using logistic regression with the AST, ALT, and GGT categories as continuous variables. Results are presented as odds ratios (*OR*s) and 95% confidence intervals (*CI*s). A value of p <0.05 was taken to indicate statistical significance. All statistical analyses were performed using PASW statistics 21 (SPSS Inc., Chicago, IL, USA).

## Results

### Baseline characteristics of subjects

Baseline characteristics of the study population according to incident diabetes status at the follow-up examination are shown in Tables 1 and 2. During the median follow-up period of 4.2 years (range 3.5 to 8.0 years), 548 subjects (235 males and 313 females) were newly identified as having type 2 diabetes.

**Table 1:** Baseline characteristics of males subjects

**Variables**	**Males (n = 2,603)**		
	**Diabetes ‡**	**Non-diabetes**	*p*-value*
Age (years)	62.4 (7.8)	62.4 (7.5)	0.848
Body mass index (kg/m^2^)	24.6 (2.9)	23.7 (2.7)	<0.001
Waist circumference (cm)	87.1 (7.6)	84.7 (7.6)	<0.001
AST (units/l)	26 (21–32)	25 (21–30)	0.089
ALT (units/l)	24 (18–34)	21 (16–29)	<0.001
GGT (units/l)	39 (24–74)	30 (19–53)	0.006
Fasting glucose (mg/dl)	108.3 (13.8)	100.1 (9.7)	<0.001
HOMA (%)	1.54 (1.10)	1.14 (1.13)	<0.001
Total cholesterol (mg/dl)	183.6 (35.9)	182.8 (34.1)	0.658
HDL cholesterol (mg/dl)	45.5 (11.7)	47.2 (12.3)	0.045
Triglycerides (mg/dl)	152 (107–231)	128 (88–194)	0.002
C-reactive protein (mg/dl)	0.08 (0.04-0.17)	0.07 (0.03-0.16)	0.757
Current drinker (%)	151 (64.3)	1,577 (66.7)	0.490
Current smoker (%)	81 (34.5)	748 (31.6)	0.548
Physical exercise (%)	87 (37.0)	819 (34.7)	0.474

Data are means (SD), medians (interquartile ranges; 25th-75th) for skewed variables, or proportions for categorical variables. *Differences were assessed using *t*-tests (for continuous variables) or *chi*-square tests (for categorical variables); Mann–Whitney tests were performed for the following skewed variables: AST, ALT, GGT, triglycerides and c-reactive protein.

‡ Diabetes status at follow-up examination.

**Table 2:** Baseline characteristics of females subjects

**Variables**	**Females (n = 4,323)**		
	**Diabetes ‡**	**Non-diabetes**	*p*-value*
Age (years)	61.7 (6.9)	60.8 (7.7)	0.049
Body mass index (kg/m^2^)	26.1 (3.9)	24.5 (3.2)	<0.001
Waist circumference (cm)	89.3 (8.7)	85.8 (8.7)	<0.001
AST (units/l)	23 (19–27)	22 (19–26)	0.076
ALT (units/l)	20 (14–26)	16 (13–21)	<0.001
GGT (units/l)	20 (15–31)	15 (12–21)	<0.001
Fasting glucose (mg/dl)	106.8 (9.6)	96.5 (9.2)	<0.001
HOMA (%)	1.74 (1.05)	1.31 (0.96)	<0.001
Total cholesterol (mg/dl)	200.2 (36.2)	192.9 (36.7)	0.001
HDL cholesterol (mg/dl)	45.8 (10.9)	48.6 (11.7)	<0.001
Triglycerides (mg/dl)	161 (113–243.5)	123 (86–177)	<0.001
C-reactive protein (mg/dl)	0.09 (0.04-0.20)	0.05 (0.02-0.11)	0.444
Current drinker (%)	109 (34.8)	1,454 (36.3)	0.112
Current smoker (%)	11 (3.5)	110 (2.7)	0.317
Physical exercise (%)	100 (31.9)	1,242 (31.0)	0.730

Data are means (SD), medians (interquartile ranges; 25th-75th) for skewed variables, or proportions for categorical variables. *Differences were assessed using *t*-tests (for continuous variables) or *chi*-square tests (for categorical variables); Mann–Whitney tests were performed for the following skewed variables: AST, ALT, GGT, triglycerides and c-reactive protein.

‡ Diabetes status at follow-up examination.

Compared to subjects who did not develop diabetes, subjects of both sexes who developed type 2 diabetes had higher BMI and WC; higher concentrations of ALT, GGT; higher fasting glucose levels, HOMA indices, and TG; and lower levels of HDL-C. Although levels of liver enzymes were within the normal range, subjects of both sexes who developed type 2 diabetes had higher concentrations of ALT, and GGT than subjects who did not develop diabetes. AST and CRP were slightly higher in those of both sexes with diabetes; however, the difference was not significant in either sex.

### AST and incidence of type 2 diabetes

There was no linear relationship between AST and incidence of type 2 diabetes. The *OR*s for incidence of diabetes according to AST quartile were 0.98 (0.63-1.52), 0.93 (0.61-1.43), and 1.07 (0.69-1.66) in males and 0.67 (0.45-0.99), 0.83 (0.57-1.12), and 0.95 (0.66-1.37) in females after adjusting for age, BMI, WC, TC, HDL-C, log-TG, alcohol intake status, smoking status, physical activity, follow-up period, log-CRP, fasting glucose, and HOMA indices (Tables 3 and 4).

**Table 3:** **Adjusted odds ratio (95**% **confidence interval) for 4-years incidence of type 2 diabetes according to quartile groups of liver enzymes (AST, ALT, and GGT) at baseline in males**

	**Q1**	**Q2**	**Q3**	**Q4**	***P *** **for trend**
AST (units/l)	≤20	21 to 24	25 to 30	≥31	
Cases/persons at risk	49/620	56/656	64/687	66/640	
Model 1	1	1.09 (0.73-1.62)	1.20 (0.81-1.77)	1.34 (0.91-1.98)	0.116
Model 2	1	1.11 (0.74-1.66)	1.10 (0.74-1.65)	1.21 (0.80-1.83)	0.390
Model 3	1	1.12 (0.73-1.71)	1.09 (0.72-1.65)	1.15 (0.76-1.76)	0.565
Model 4	1	0.98 (0.63-1.52)	0.93 (0.61-1.43)	1.07 (0.69-1.66)	0.794
ALT (units/l)	≤15	16 to 20	21 to 29	≥30	
Cases/persons at risk	31/615	46/632	81/710	77/646	
Model 1	1	1.49 (0.93-2.39)	2.49 (1.62-3.83)	2.67 (1.72-4.15)	<0.001
Model 2	1	1.42 (0.89-2.28)	2.19 (1.41-3.41)	2.11 (1.33-3.35)	<0.001
Model 3	1	1.53 (0.93-2.52)	2.24 (1.40-3.59)	2.31 (1.42-3.75)	<0.001
Model 4	1	1.36 (0.82-2.27)	2.03 (1.26-3.29)	1.95 (1.18-3.21)	0.004
GGT (units/l)	≤19	20 to 30	31 to 54	≥55	
Cases/persons at risk	38/661	44/651	72/635	81/656	
Model 1	1	1.20 (0.77-1.89)	2.14 (1.42-3.23)	2.38 (1.59-3.57)	<0.001
Model 2	1	1.09 (0.69-1.73)	2.00 (1.28-3.12)	2.13 (1.33-3.41)	<0.001
Model 3	1	1.00 (0.62-1.63)	1.86 (1.17-2.96)	1.97 (1.21-3.22)	0.001
Model 4	1	0.86 (0.52-1.42)	1.57 (0.97-2.54)	1.58 (0.95-2.63)	0.011

Model 1: adjusted for age.

Model 2: Model 1 plus body mass index, waist circumference, total cholesterol, HDL-cholesterol, log-triglyceride, alcohol intake status, smoking status, physical activity, and follow-up period.

Model 3: Model 2 plus log-CRP.

Model 4: Model 3 plus fasting glucose and HOMA.

**Table 4:** **Adjusted odds ratio (95**% **confidence interval) for 4-years incidence of type 2 diabetes according to quartile groups of liver enzymes (AST, ALT, and GGT) at baseline in females**

	**Q1**	**Q2**	**Q3**	**Q4**	***P *** **for trend**
AST (units/l)	≤18	19 to 21	22 to 25	≥26	
Cases/persons at risk	74/983	58/1,066	86/1,187	95/1,104	
Model 1	1	0.69 (0.48-0.98)	0.92 (0.67-1.28)	1.11 (0.80-1.52)	0.212
Model 2	1	0.66 (0.46-0.95)	0.87 (0.62-1.22)	0.99 (0.71-1.38)	0.567
Model 3	1	0.72 (0.49-1.05)	0.88 (0.62-1.25)	1.01 (0.72-1.43)	0.562
Model 4	1	0.67 (0.45-0.99)	0.83 (0.57-1.12)	0.95 (0.66-1.37)	0.783
ALT (units/l)	≤13	14 to 16	17 to 21	≥22	
Cases/persons at risk	56/1,199	52/994	77/1,003	128/1,127	
Model 1	1	1.12 (0.76-1.65)	1.69 (1.18-2.40)	2.65 (1.91-3.67)	<0.001
Model 2	1	0.99 (0.67-1.47)	1.40 (0.97-2.01)	1.86 (1.32-2.61)	<0.001
Model 3	1	0.97 (0.64-1.48)	1.38 (0.94-2.02)	1.86 (1.31-2.65)	<0.001
Model 4	1	0.86 (0.56-1.32)	1.23 (0.83-1.83)	1.49 (1.03-2.16)	0.006
GGT (units/l)	≤12	13 to 15	16 to 21	≥22	
Cases/persons at risk	45/1,248	42/882	85/1,076	141/1,117	
Model 1	1	1.35 (0.88-2.07)	2.30 (1.59-3.33)	3.88 (2.74-5.49)	<0.001
Model 2	1	1.14 (0.74-1.77)	1.73 (1.18-2.54)	2.69 (1.86-3.89)	<0.001
Model 3	1	1.11 (0.70-1.76)	1.60 (1.06-2.39)	2.45 (1.66-3.61)	<0.001
Model 4	1	0.97 (0.60-1.55)	1.27 (0.83-1.94)	1.85 (1.23-2.79)	<0.001

Model 1: adjusted for age.

Model 2: Model 1 plus body mass index, waist circumference, total cholesterol, HDL-cholesterol, log-triglyceride, alcohol intake status, smoking status, physical activity, and follow-up period.

Model 3: Model 2 plus log-CRP.

Model 4: Model 3 plus fasting glucose and HOMA.

### ALT and incidence of type 2 diabetes

After adjusting for age, BMI, WC, TC, HDL-C, log-TG, alcohol intake status, smoking status, physical activity, follow-up period, and log-CRP, the *OR*s for incidence of diabetes according to ALT quartile were 1.53, 2.24, and 2.31 in males and 0.97, 1.38, and 1.86 in females (Tables 3 and 4). Further adjustment for fasting glucose and HOMA indices attenuated the association; however, ALT remained a significant risk factor in both sexes. Comparison of the highest versus the lowest quartiles for ALT showed *OR*s for incidence of type 2 diabetes of 1.95 (95% *CI*: 1.18-3.21) in males and 1.49 (95% *CI*: 1.03-2.16) in females. Interactions of ALT with alcohol intake status, BMI and WC were not significant in either sex (data not shown).

### GGT and incidence of type 2 diabetes

According to all models, there was a positive linear relationship between GGT and incidence of type 2 diabetes in both sexes (Tables 3 and 4). After adjusting for age, BMI, WC, TC, HCL-C, log-TG, alcohol intake status, smoking status, physical activity, follow-up period, and log-CRP, the *OR*s for incidence of diabetes according to GGT quartile were 1.00, 1.86, and 1.97 in males and 1.11, 1.60, and 2.45 in females (Tables 3 and 4). Further adjustment for fasting glucose and HOMA indices attenuated the association; however, GGT remained a significant risk factor in females. When the fourth quartile of GGT was compared with the first quartile, *OR*s for incidence of diabetes were 1.58 (95% *CI*: 0.95-2.63) in males and 1.85 (95% *CI*: 1.23-2.79) in females. Interactions of GGT with alcohol intake status, BMI and WC were not significant in either sex (data not shown).

## Discussion

This study examined the associations of serum liver enzymes on the incidence of type 2 diabetes in a prospective study of a general Korean population. Our results suggest that serum ALT and GGT were positively associated with an increased risk of type 2 diabetes in both sexes, and no interactions were seen between ALT or GGT and alcohol intake status, BMI, or WC. After adjustment for potential confounders, we found that elevated levels of ALT were independently associated with type 2 diabetes in both sexes, with an independent association for GGT only in females.

In the present study, after adjusting for diabetes risk factors, the association between AST and incidence of diabetes was not significant in either sex. Our results are consistent with those reported by Nakanishi et al. [21], who found no association of AST with diabetes risk after adjustment for age, family history of diabetes, BMI, alcohol intake, cigarette smoking, physical activity, systolic blood pressure, lipid profile, fasting glucose, and white blood cell count in a study of male Japanese office workers. In contrast, in the prospective The Insulin Resistance Atherosclerosis Study, Hanley et al. [5] reported that AST independently predicted type 2 diabetes after adjustment for covariates, including metabolic syndrome variables, directly measured insulin sensitivity, acute insulin response, and CRP. The *OR* of type 2 diabetes for the highest versus the lowest quartiles was 1.98 (95% *CI*: 1.23-3.17). Although AST is a marker of hepatocellular health, it is a less specific marker of liver function than ALT and GGT [4]. Therefore, AST may be a less specific marker of liver pathology related to development of type 2 diabetes.

Some prospective studies [5,8,10,11,13,14,24] have reported that higher ALT concentrations predicted development of type 2 diabetes. Our data also indicated that ALT was an independent association in both sexes after adjusting for other diabetes risk factors. In the late 1980s, Ohlson et al. [25] reported that baseline ALT was a predictor of the incidence of type 2 diabetes after 13.5 years of follow-up in a cohort of 766 Swedish males, with a significant fourfold increased risk for males in the upper quintile compared to the lowest quintile.

Serum GGT has been widely used as an index of liver dysfunction and as a biological marker of alcohol abuse [17,21,26]. Most previous studies [3,4,6,7,10,12,14,16-20] reported an association of GGT concentrations with the incidence of type 2 diabetes, and our results are in agreement with these findings. A 4-year follow-up prospective cohort study in a Korean population [4] showed a strong dose–response relationship between the serum GGT concentration at baseline and the incidence of type 2 diabetes. However, two studies reported no association of GGT concentration with the development of type 2 diabetes [8,25]. In a longitudinal study of Pima Indians [8], despite a strong correlation between ALT and GGT concentrations, only ALT was positively associated with the risk of type 2 diabetes. In the Pima Indian study, alcohol consumption was not evaluated, which could have obscured the association between GGT and development of type 2 diabetes. In our study, as in others, the association of GGT with the incidence of type 2 diabetes was independent of alcohol consumption [3,4,6,17,18,21].

Although the mechanism underlying the associations between liver enzymes and incidence of type 2 diabetes remains unclear, some possibilities can be considered. One is that increased serum AST, ALT, and GGT levels reflect an excess deposit of fat in the liver, a condition known as non-alcoholic fatty liver disease (NAFLD). NAFLD is considered a hepatic manifestation of metabolic syndrome, which refers to a cluster of cardiovascular risk factors associated with insulin resistance, including central obesity, hypertension, dyslipidemia, and type 2 diabetes [27]. NAFLD, which is closely related to obesity and visceral fat deposition, is now regarded as a feature of insulin resistance syndrome [2], and visceral adipose tissue is known to confer a significantly higher risk of type 2 diabetes [28]. Excess visceral fat accumulation may be causally related to features of insulin resistance as well as to high plasma levels of insulin and glucose [29,30]. Overt diabetes is thought to be preceded by a long period of insulin resistance, during which blood glucose is maintained at a near-normal level by compensatory hyperinsulinemia [31]. In this study, ALT concentrations were independently associated with the incidence of type 2 diabetes in both sexes, after adjusting for HOMA as a marker of insulin resistance and fasting glucose, whereas GGT was independently associated only in females.

A second possibility is that GGT plays an important role in antioxidant systems, with the primary function of maintaining intracellular glutathione levels, a major intracellular antioxidant defense for the cell [32-34]. Increased oxidative stress contributes to the development and progression of diabetes [35,36] and chronic oxidative stress results in decreased responsiveness to insulin, ultimately leading to type 2 diabetes [30]. Although the mechanism remains largely unknown, there is clear evidence that cellular GGT concentration is closely related to oxidative stress indicators *in vivo*[37-39]. Inflammation has also been recognized as a manifestation of oxidative stress, and all pathways that generate mediators of inflammation, such as adhesion molecules and interleukins, are induced by oxidative stress [40]. Several prospective studies have demonstrated that elevated CRP levels were significantly predictive of risk of type 2 diabetes [41-46]. Changes in inflammation that occur through oxidative stress are assumed to be a common step in the pathogenesis of type 2 diabetes. In the present study, after adjusting for CRP as a marker of inflammation and/or oxidative stress, GGT was found to be a significantly associated with the incidence of diabetes in both sexes. These results suggested that the association was independent of inflammation and oxidative stress.

The present study had the following limitation. We were unable to complete follow-up examinations for all participants who were still alive at the time of re-examination, which may have introduced a selection bias.

The strengths of this study relate primarily to its prospective design, with inclusion of a large sample size and availability of data on lifestyles and diabetes risk factors. Second, in contrast to most other prospective studies, in which the diagnosis of diabetes was based only on self-reporting, the diagnosis of diabetes in the present study was based on both self-reporting and biological markers. Third, we used relevant information to exclude subjects with various etiologies of liver pathology, such as viral hepatitis, liver cirrhosis, and liver cancer. Fourth, our study considered the HOMA index, which is a well-validated method for measurement of insulin resistance and has shown a moderate-to-strong correlation (0.58 to 0.88) with reference techniques, such as the euglycemic clamp [47].

## Conclusions

In conclusion, in this prospective study, over a median period of 4.2 years and after adjusting for potential confounders, elevated serum ALT concentrations in both sexes and GGT only in females were independently associated with type 2 diabetes. The associations between these enzymes and incidence of type 2 diabetes were independent of insulin resistance, inflammation and/or oxidative stress markers. Although the mechanisms underpinning these associations require further investigation, these results support the hypothesis that the liver is important in the pathogenesis of type 2 diabetes and that hepatic enzymes may be useful additional markers of subjects at high risk for development of diabetes.

## Abbreviations

AST: Aspartate aminotransferase; ALT: Alanine aminotransferase; GGT: Gamma-glutamyltransferase; BMI: Body mass index; WC: Waist circumference; TC: Total cholesterol; HDL-C: High-density lipoprotein cholesterol; TG: Triglycerides (TG); CRP: C-reactive protein; HOMA: Homeostasis model assessment; NAFLD: Non-alcoholic fatty liver disease.

## Competing interests

The authors declare no competing interests.

## Authors’ contributions

HR prepared the study proposal, collected data in the field, analyzed the data and drafted the manuscript. SS and MH contributed to the study design, collected data in the field, assisted with the preparation of the proposal, and contributed to the manuscript. HS, KS, YH, and SK collected data in the field, interpretation of data and reviewed manuscript. JS interpretation of data and critically reviewed the manuscript. All authors read and approved the final manuscript.
